# Human Immunodeficiency Virus (HIV) Knowledge and Stigma: Findings from a Cross-sectional Survey

**DOI:** 10.21106/IJMA_7_2025

**Published:** 2026-01-29

**Authors:** Wah Wah Myint

**Affiliations:** 1 Center for Community Health and Aging, School of Public Health, Texas A&M University, Texas, United States.

**Keywords:** Antiretroviral Drugs, Human Immunodeficiency Virus Stigma, Human Immunodeficiency Virus Test Kits, Human Immunodeficiency Virus Transmission and Risk Reduction, The Philippines

## Abstract

**Background and Objective:**

People living with human immunodeficiency virus (HIV) encounter HIV stigma that hinders HIV testing and subsequently linkage to care. This study evaluates the relationship between HIV stigma and awareness of HIV testing, drugs to prevent mother-to-child transmission (MTCT), antiretroviral (ARV) drugs, and HIV knowledge score among Filipino women.

**Methods:**

We utilized data of women aged 15–49 (*n* = 25,284) who heard about HIV from the Philippines’ 2022 Demographic and Health Survey. The dependent variable was HIV stigma. The covariates were sociodemographic characteristics (i.e., age, urban/rural, education level, wealth quintile, current employment status, and marital status), knowledge regarding HIV test kits, drugs to prevent MTCT, and ARV drugs. We calculated descriptive statistics, Pearson’s Chi-square test of independence, and multivariable binary logistic regression, fitting covariates. Subgroup analysis was performed to understand the relationship between HIV stigma and HIV knowledge score among individuals aged 15–24 years. All analyses were performed using Stata 18.5.

**Results:**

Approximately 65.7% of women reported HIV stigma. The logistic regression results indicated lower odds of HIV stigma among women who know test kits but never tested for HIV with test kits (adjusted odds ratio [aOR] = 0.87), those who heard about drugs to prevent MTCT (aOR = 0.75), and those who heard about ARV drugs (aOR = 0.85) compared to their counterparts. Higher odds of HIV stigma were observed among women currently in union (aOR = 1.46) and rural residents (aOR = 1.54) compared to their counterparts. Among women aged 15–24, having a higher HIV knowledge score, especially among those who scored 5, had lower odds of having HIV stigma (aOR = 0.04) than those who achieved a zero knowledge score. All *p*-values ≤ 0.05.

**Conclusion and Global Health Implications:**

Findings suggest an urgent need to address stigma among women, especially those who lack knowledge of HIV test kits and ARV drugs. Further research should explore strategies to reduce HIV stigma, especially among young married rural women.

## INTRODUCTION

Human immunodeficiency virus (HIV) is one of the behavioral diseases, as its main transmission routes include sexual contact, mother-to-child transmission (MTCT), and sharing needles and syringes.

### Background of the Study

In 2023, the global estimate of people living with HIV (PLHIV) was around 39.9 million, of which 4 million resided in the Southeast Asia (SEA) region.^[1]^ Despite progress, some countries in the SEA region showed an increasing trend. For instance, the Philippines showed the fastest-growing HIV rate with over 160 thousand PLHIV.^[1]^ Often, an individual who required testing was hindered by the HIV stigma (i.e., having negative attitudes and beliefs toward HIV and PLHIV).^[2]^

The HIV stigma manifests in various forms. It could be social stigma (e.g., negative attitudes toward PLHIV) or enacted stigma (e.g., specific discriminatory acts toward PLHIV). It could also be internalized stigma (e.g., individuals’ beliefs and acceptance of negative social beliefs) or anticipated stigma (e.g., fear of a potential stigmatizing attitude).^[3]^ Prior research revealed that HIV stigma caused adverse health impacts, including depression, post-traumatic stress disorder, and engagement in risky health behaviors.^[4,5]^ Literature also indicated that youth and women may encounter a greater extent of HIV stigma.^[5]^ The HIV stigma may exist both within families and in broader contexts, including community and healthcare environments.^[6]^ Stigma is a significant barrier for testing, care and treatment, adherence, and attainment of undetectable or untransmissible (U=U) viral loads.^[7,8]^ The attainment of U=U is important because it helps to end the HIV pandemic by preventing HIV transmission.^[7]^

Specifically, evidence indicated that PLHIV who experienced HIV stigma were 2.4 times more likely to delay enrollment in care and treatment and had decreased average survival time.^[9,10,11]^ Some studies also found a linkage between HIV stigma and reduced adherence to antiretroviral (ARV) drugs, decreased utilization of health and social services, increased rate of depression, and cognitive performance decline.^[12,13,14]^ Many efforts have been made to reduce HIV stigma. For example, the Joint United Nations Programme on HIV/AIDS has set the 10-10-10 targets for social enablers to be achieved by 2025, which includes a goal to reduce stigma and discrimination against PLHIV by 10%.^[11]^ However, this target remains distant and may not be achieved by 2025, unless the countries take immediate action.^[13,15]^

#### HIV Situation in the Philippines

The 2023 data indicated that about 95% of the reported HIV cases in the Philippines were males, specifically highest among those aged 15–24 years.^[16]^ The predominant mode of transmission was through sexual contact.^[16]^ From 2018 to 2023, HIV incidence among women increased by 91%.^[16]^ The high incidence among women highlights a need for immediate action to ensure women receive early HIV tests. Furthermore, there is a need to understand the underlying factors, including stigma, that deter them from HIV testing.

#### Prior literature regarding HIV stigma among Filipino individuals

Prior studies in the Philippines have had inconsistent findings regarding stigma.^[17,18,19]^ And their focus populations were males who have sex with males; transgender women; or cisgender men^[17]^; PLHIV^[18]^; women living with HIV^[18]^; and healthcare workers^[19]^, lacking a focus on HIV stigma among the general women population.^[20]^ Moreover, there exists a paucity of literature regarding stigma among women related to their knowledge of HIV test kits and HIV medication, despite extensive research concentrating on HIV prevention and transmission.

### Objectives of the Study

This study aims to examine the relationship between HIV stigma and sociodemographic characteristics and HIV-related knowledge.

### Speciflc Aims and Hypothesis

The aim will be reached through three main objectives: (1) To describe the prevalence of knowledge on HIV test kits and HIV medications, (2) to examine the relationship between HIV stigma and HIV knowledge about HIV test kits and medication among women aged 15–49, and (3) to examine the relationship between HIV stigma and knowledge of HIV prevention and risk reduction among women aged 15–24.

## METHODS

De-identifiable data (*n* = 27,821) of women aged 15–49 from the Philippines’ 2022 National Demographic and Health Survey were used.^[21]^ The cross-sectional survey applied two-staged stratified sampling aiming to reduce the bias, and it was collected in the Philippines from May 2 to June 22, 2022.^[21]^ The women who reported that they had heard about HIV/AIDS were included in the study. Of the total respondents aged 15–49, 90.9% (*n* = 25,284) of women reported that they had heard about HIV. For the HIV transmission and risk reduction knowledge, we used a separate sub-population analysis because the data are available only for the women aged 15–24 (*n* = 10,388). As recommended by the Enhancing the Quality and Transparency of Health Research Network, we followed the Strengthening the Reporting of Observational studies in Epidemiology’s reporting guideline for cross-sectional studies.^[22]^

### Study Variables

#### Outcome variable

The outcome variable was HIV stigma, a binary variable. It is a composite variable created by combining two available HIV stigma measures. The first question asked: “Should children with HIV be allowed to attend the same school as children who do not have HIV?” and the responses were “no” or “yes.” The second question asked: “Would you buy vegetables from a vendor with HIV?” And the responses were “no,” and “yes.” If a respondent said “yes” to both questions, it is recoded as “no stigma” and “no” to either of those questions, it is recoded as “having HIV stigma.” These two indicators are aligned with the UNAID’s Global AIDS Monitoring (GAM) indicator 6.1 on discriminatory attitudes.^[11]^

#### Sociodemographic variables

The sociodemographic variables included age groups in 5-year interval (15–19, 20–24, 25–29, 30–34, 35–39, 40–44,

45–49), education level (no education, primary, secondary, higher), marital status (never in union, currently in union/ living with a man, formerly in union/living with a man), current employment (no, yes), wealth quintile (poorest, poorer, middle, richer, richest), and place of residency (urban, rural).

#### Other covariates

Knowledge on HIV test kits and medication among women 15–49

This study also included four main covariates that asked about knowledge of HIV test kits and drugs for HIV prevention and treatment. The knowledge of HIV test kits is a categorical variable with response options “never heard about it,” “has tested for HIV,” and “knows test results.” Knowledge regarding drugs for HIV prevention and treatment was: If the women had heard of ARV drugs that treat HIV (no, yes), and if the women knew that the risk of MTCT can be reduced by the mother taking special drugs (no, yes).

HIV knowledge on transmission and risk reduction among women aged 15–24

**Figure 1:**
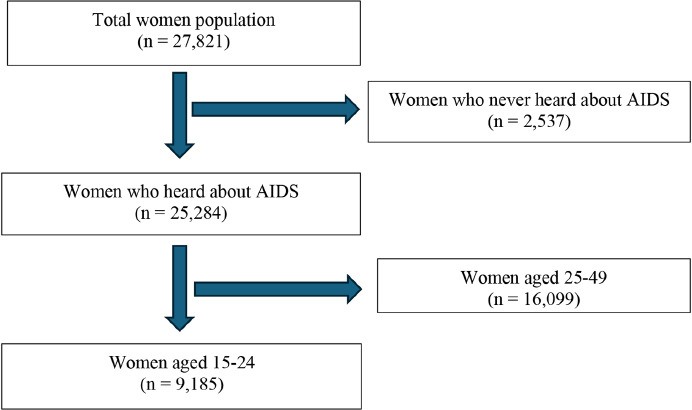
Sample flow Chart

The DHS 2022 asked comprehensive HIV prevention knowledge only to women aged 15–24 years, and therefore, we did a separate subgroup analysis for the 15–24 age group (*n* = 10,388). For our final sample, a screening question was asked: “Ever heard of HIV?” with the responses “yes” or “no.” We excluded 1,203 (11.6%) who had not heard about HIV, leaving our final sample to 9,185 (88.4%) who reported and heard about HIV among women aged 15–24 [Figure 1].

The HIV knowledge score on transmission and risk reduction (hereafter, we will refer to this as the HIV knowledge score) is a composite variable (score range = 0–5), which is created from five knowledge variables. It included two HIV risk reduction variables (i.e., know that using a condom every time they have sex can reduce the risk of getting HIV, and know that having just one uninfected faithful partner can reduce the risk of getting HIV). In addition, two misconceptions of HIV transmission variables (i.e., knowing that HIV cannot be transmitted by mosquito bites, knowing that a person cannot become infected by sharing food with a person who has HIV) and one misconception that a healthy-looking person can have HIV were included. If a person responded each question correctly, we assigned “1,” and if the person answered incorrectly, we assigned “0,” respectively. The reference groups were chosen based on prior literature.

### Statistical Analysis

We performed univariate analyses to observe the frequency and percentage of included variables. Bivariate analyses, i.e., Pearson’s Chi-square test of independence, were conducted to assess whether the included variables exhibit a significant relationship with HIV stigma. Two multivariable binary logistic regression models (one with all women aged 15–49 years and the other model only with women aged 15–24 years) were performed to predict the HIV stigma, fitting all the covariates. The analyses were applied to survey weights and conducted using Stata 18.5 version.^[23]^ Missingness 10% was reported (if any). P-value <0.05 is considered significant.

### Ethical Approval

The DHS survey has obtained ethical approval from the Inner-City Fund Institutional Review Board, which was carried out in compliance with regulations for the protection of human subjects (45 Code of Federal Regulation 46) of the U.S. Department of Health and Human Services.^[21]^

## RESULTS

Among the women aged 15–49, about 48% were not aware that there were medications to prevent MTCT during pregnancy, and almost 70% had not heard about ARV drugs to treat HIV. Moreover, many respondents (79%) never heard about HIV test kits, nor ever used them (21%). Overall, 65.7% had at least one HIV stigma. Among the women aged 18– 24 years, about 21%–40% had at least one misconception on transmission or risk reduction questions. For example, about 22% still responded that HIV can get through mosquito bites, or do not know.

### Findings from Bivariate Analysis

We present the bivariate analysis results of HIV stigma and the covariates [Table 1]. A higher proportion of HIV stigma was seen among women aged 15–18 (χ^2^ = 143.1, *p* < 0.001), living in urban (χ^2^ = 712.1, *p* < 0.001), those who had secondary education (χ^2^ = 984.4, *p* < 0.001), those who were from the middle wealth quintiles groups (χ^2^ = 1268.0, *p* < 0.001), currently in union (χ^2^ = 136.4), unemployed (χ^2^ = 155.4, *p* < 0.001). Furthermore, a larger proportion of stigma was observed among those who had no knowledge regarding HIV test kits (χ^2^ = 219.3, *p* < 0.001) and drugs that prevent MTCT (χ^2^ = 200.9, *p* < 0.001), and ARV drugs (χ^2^ = 202.8, *p* < 0.001). Among the youth, a larger proportion of stigma was observed among youth who answered correctly on knowledge questions such as risk reduction by condom use (χ^2^ = 326.1, *p* < 0.001), having only one partner (χ^2^ = 70.3, *p* < 0.001), and transmission by mosquito bites (χ^2^ = 181.1, *p* < 0.001), and a healthy-looking person can have HIV (χ^2^ = 200.3, *p* < 0.001). A larger proportion of youth incorrectly answered that one can get HIV by sharing food with a PLHIV (χ^2^ = 926.7, *p* < 0.001). Among the youth, a greater proportion of stigma was seen among those who had four correct answers (χ^2^ = 1109.0, *p* < 0.001) [Table 1].

**Table 1:** Bivariate results of the presence of HIV stigma and covariates (column percentage).

All women aged 15–49					
Sociodemographic characteristics	No stigma: 7,518	Stigma: 17,766	Total: 25,284	χ^2^	*p*-value
	*n* (%)	*n* (%)	*n* (%)		
Age groups					
15–19	1,259 (14.9)	3,714 (20.1)	4,973 (18.3)	143.1	<0.001
20–24	1,447 (19.7)	2,765 (15.8)	4,212 (17.2)		
25–29	1,045 (15.2)	2,446 (14.0)	3,491 (14.4)		
30–34	1,049 (13.9)	2,320 (13.5)	3,369 (13.6)		
35–39	923 (12.5)	2,211 (12.6)	3,134 (12.6)		
40–44	975 (12.8)	2,188 (12.3)	3,163 (12.5)		
45–49	820 (11.0)	2,122 (11.7)	2,942 (11.5)		
Education level					
No education	19 (0.3)	96 (0.4)	115 (0.4)	984.4	<0.001
Primary	357 (4.1)	1,990 (10.1)	2,347 (8.0)		
Secondary	3,355 (43.9)	9,769 (57.0)	1,3124 (52.5)		
Higher	3,787 (51.8)	5,911 (32.5)	9,698 (39.1)		
Marital status					
Never in union	3,385 (45.5)	6,796 (38.2)	10,181 (40.7)	136.4	<0.001
Currently in union	3,869 (50.9)	10,390 (58.5)	14,259 (55.9)		
Formerly in union	264 (3.6)	580 (3.3)	844 (3.4)		
Current employment					
No	3,728 (48.1)	10,125 (56.3)	1,3853 (53.5)	155.4	<0.001
Yes	3,790 (51.9)	7,641 (43.7)	11,431 (46.5)		
Wealth quintile					
Poorest	832 (7.22)	4,497 (18.6)	5,329 (14.7)	1,268.0	<0.001
Poorer	1,308 (13.58)	4,245 (21.2)	5,553 (18.6)		
Middle	1,487 (20.01)	3,428 (21.1)	4,915 (20.8)		
Richer	1,751 (26.01)	2,938 (20.4)	4,689 (22.3)		
Richest	2,140 (33.18)	2,658 (18.7)	4,798 (23.7)		
Place of residency					
Urban	4,037 (68.5)	6,540 (51.0)	10,577 (57.0)	712.1	<0.001
Rural	3,481 (31.5)	11,226 (49.0)	14,707 (43.0)		
Knowledge and use of HIV test kits					
Never heard of HIV test kits	5,647 (73.37)	14,758 (81.39)	20,405 (78.64)	219.3	<0.001
Has been tested with HIV test kits	74 (1.01)	85 (0.60)	159 (0.74)		
Knows HIV test kits but has never tested	1,797 (25.61)	2,923 (18.01)	4,720 (20.62)		
Drugs to avoid HIV transmission to the baby during pregnancy					
No	3,078 (41.45)	9,013 (50.83)	12,091 (47.61)	200.9	<0.001
Yes	4,440 (58.55)	8,753 (49.17)	13,193 (52.39)		
Heard of ARV drugs to treat HIV					
No	4,932 (64.70)	13,103 (73.32)	18,035 (70.36)	202.8	<0.001
Yes	2,586 (35.30)	4,663 (26.68)	7,249 (29.64)		
Reduce the risk of getting HIV: Always use a condom during sex				χ^2^	*p*-value
Incorrect response	492 (15.86)	2,332 (33.37)	2,824 (27.51)	314.5	<0.001
Correct response	2,214 (84.14)	4,147 (66.63)	6,361 (72.49)		
Reduce risk of getting HIV: Have one sex partner only, who has no other partners					
Incorrect response	487 (18.28)	1,760 (26.14)	2,247 (23.51)	70.2	<0.001
Correct response	2,219 (81.72)	4,719 (73.86)	6,938 (76.49)		
Can get HIV from mosquito bites					
Incorrect response	392 (13.64)	1,823 (25.92)	2,215 (21.81)	73.8	<0.001
Correct response	2,314 (86.36)	4,656 (74.08)	6,970 (78.19)		
Can get HIV by sharing food with a person who has AIDS					
Incorrect response	520 (18.02)	3,236 (51.05)	3,756 (40.02)	926.7	<0.001
Correct response	2,186 (81.93)	3,243 (48.95)	5,429 (59.98)		
Respondent thinks that a healthy-looking person can have HIV					
Incorrect response	361 (12.86)	1,804 (25.69)	2,165 (21.40)	200.3	<0.001
Correct response	2,345 (87.14)	4,675 (74.31)	7,020 (78.60)		
HIV knowledge score					
Zero score	10 (0.13)	159 (1.77)	169 (1.22)	1,109.0	<0.001
Score 1	33 (1.42)	403 (5.58)	436 (4.19)		
Score 2	128 (4.20)	1,027 (14.79)	1,155 (11.25)		
Score 3	411 (14.15)	1,780 (28.03)	2,191 (23.39)		
Score 4	864 (31.48)	1,907 (30.57)	2,771 (30.87)		
Score 5	1,260 (48.63)	1,203 (19.26)	2,463 (29.09)		

HIV: Human immunodeficiency virus, AIDS: Acquired immune deficiency syndrome, ARV: Antiretroviral. *p*-value <0.05 is considered significant.

### Findings from Multinomial Binary Logistic Regression Results

The findings of the logistic regression analysis among women aged 15–49 were displayed in Table 2. Overall, women from the older age groups, those in current employment, being in the richest wealth quintile, knowledge of HIV test kits, and HIV drugs were strong predictors for no HIV stigma, while those who were currently in union and those living in rural areas were strong predictors for HIV stigma. Among the age groups, the lowest odds of HIV stigma was seen among women aged 40–44 (adjusted odds ratio [aOR] = 0.72, *p* = 0.001), those who had current employment (aOR = 0.90, *p* < 0.021), those from the richest wealth quintile groups (aOR = 0.42, *p* < 0.001), those who knows test kits but never tested HIV with test kits (aOR = 0.87, *p* = 0.015), those who heard about HIV drugs to prevent MTCT during pregnancy (aOR = 0.75, *p* < 0.001), those who heard about ARV drugs to treat HIV (aOR = 0.85, *p* < 0.001) compared to the reference groups [Table 2]. There were higher odds of HIV stigma among those who were currently in union (aOR = 1.46, *p* < 0.001) and rural (aOR = 1.54, *p* < 0.001).

**Table 2:** Multivariable logistic regression results: Relationship between HIV-related stigma and covariates (sociodemographic characteristics, HIV testing, and medication knowledge) among women aged 15–49.

Covariates	aOR (95% CI)	*p*-value
Age group		
15–19	Reference	
20–24	0.76 (0.64–0.90)	0.002
25–29	0.81 (0.67–0.99)	0.04
30–34	0.74 (0.61–0.91)	0.005
35–39	0.74 (0.61–0.91)	0.004
40–44	0.72 (0.59–0.87)	0.001
45–49	0.76 (0.61–0.94)	0.012
Education level		
No education	Reference
Primary	1.51 (0.77–2.93)	0.230
Secondary	1.10 (0.57–2.11)	0.783
Higher	0.75 (0.39–1.45)	0.393
Marital status		
Never in union	Reference
Currently in union/living with a man	1.46 (1.29–1.65)	<0.001
Formerly in union/living with a man	1.23 (0.97–1.56)	0.084
Current employment		
No	Reference
Yes	0.90 (0.82–0.98)	0.021
Wealth quintile		
Poorest	Reference
Poorer	0.74 (0.64–0.87)	<0.001
Middle	0.61 (0.51–0.72)	<0.001
Richer	0.52 (0.44–0.62)	<0.001
Richest	0.42 (0.35–0.50)	<0.001
Place of residency		
Urban	Reference
Rural	1.54 (1.45,1.63)	<0.001
Knowledge and use of HIV test kits		
Never heard and use of HIV test kits	Reference
Has been tested with HIV test kits	0.72 (0.47–1.10)	0.132
Knows test kits but has never tested with test kits	0.87 (0.78–0.97)	0.015
Drugs to prevent MTCT		
No	Reference
Yes	0.75 (0.68–0.83)	<0.001
Heard of ARV drugs to treat HIV		
No	Reference
Yes	0.85 (0.77–0.93)	0.001
Constant	3.80 (1.98–7.31)	<0.001

aOR: Adjusted odds ratio, 95%CI: 95% confidence interval, HIV: Human immunodeficiency virus, ARV: Antiretroviral, MTCT: Mother-to-child transmission. P-value <0.05 is considered significant.

### Findings from Multinomial Binary Logistic Regression Results of Sub-group Analysis

The results from the subgroup analysis [Table 3] showed that women who were currently in union (aOR = 1.45, *p* < 0.001) and living in rural areas (aOR = 1.60, *p* < 0.001) had higher odds of HIV stigma than their counterparts. Contrarily, those who were from the richest quintiles (aOR = 0.46, *p* < 0.001) and those who had one or more HIV knowledge scores had lower odds compared to their counterparts. The lowest odds ratio was observed among those who had an HIV knowledge score of 5 (aOR = 0.04, *p* < 0.001) compared to those who had a zero HIV knowledge score.

**Table 3:** Multivariable logistic regression results: HIV stigma and covariates (sociodemographic variables and HIV knowledge on transmission and risk reduction) among women aged 15–24.

Covariates	aOR (95% CI)	*p*-value
Education level		
No education	Reference	
Primary	1.57 (0.48–5.09)	0.452
Secondary	2.43 (0.82–7.19)	0.108
Higher	1.55 (0.52–4.64)	0.437
Marital status		
Never in union	Reference	
Currently in union	1.45 (1.17–1.80)	0.001
Formerly in union	1.59 (0.75–3.36)	0.227
Current employment		
No	Reference	
Yes	0.90 (0.76–1.08)	0.267
Wealth quintile		
Poorest	Reference	
Poorer	0.64 (0.49–0.83)	<0.001
Middle	0.60 (0.46–0.79)	<0.001
Richer	0.55 (0.42–0.72)	<0.001
Richest	0.46 (0.35–0.61)	<0.001
Place of residence		
Urban	Reference	
Rural	1.60 (1.36–1.8)	<0.001
HIV knowledge score		
Zero score	Reference	
Score 1	0.31 (0.10–0.90)	0.032
Score 2	0.27 (0.10–0.68)	0.005
Score 3	0.16 (0.06–0.40)	<0.001
Score 4	0.08 (0.03–0.20)	<0.001
Score 5	0.04 (0.01–0.09)	<0.001
Constant	15.12 (3.64–62.87)	<0.001

aOR: Adjusted odds ratio, 95%CI: 95% confidence interval, HIV: Human immunodeficiency virus. P-value <0.05 is considered significant.

## DISCUSSION

The aim of the study was to evaluate the relationship between HIV stigma and the socio-demographic characteristics and other important covariates among Filipino women. The first objective [Table 1], the second objective [Table 2], and the third objective [Table 3] have been achieved as follows.

As the current study demonstrated, 65.7% of women aged 15–49 years reported having at least one form of HIV stigma. This finding highlighted that there is an urgent need to reduce HIV stigma. A multicounty study revealed that nearly 95% of survey participants answered that they would buy vegetables from the vendor with HIV, while our findings identified only 57%.^[24]^ This represents a concerning stigmatizing attitude that necessitates immediate actions to mitigate HIV stigma toward PLHIV, since such stigma may hinder timely access to care and treatment.^[25]^

Moreover, data from the 2023 ART registry of the Philippines’ Department of Health indicate that 24% of 2,000 newly reported cases were diagnosed with advanced HIV clinical manifestations, suggesting that HIV stigma may contribute to delayed diagnosis.^[16]^ Our findings corroborated this assumption. A lack of knowledge on HIV test kits and an elevated HIV stigma may contribute to delays in diagnosis and enrollment in care and treatment.

While our analysis lacked data on HIV stigma within health facilities, a study conducted in the Philippines revealed that a considerable proportion of PLHIV experienced HIV stigma in seeking health services, necessitating further research.^[25]^ One of the promising strategies could be optimizing HIV services for HIV-infected and at-risk populations within the public sector of the Philippines, including service provider sensitization training and the assurance of service confidentiality.^[26]^

Our findings from logistic regression among women aged 15–49 regarding no substantial link between HIV stigma and education contradict a prior study’s findings.^[27]^ Naz (2019) found that individuals with a higher educational status experienced reduced HIV stigma compared to their counterparts. However, our finding regarding the fact that women who were currently in union had a higher HIV stigma supports the findings of Naz (2019), which found that married women exhibit a higher HIV stigma.^[27]^ The mechanism behind the higher HIV stigma among married women was unknown. It is possible that married women, especially in a monogamous society, may link HIV with infidelity and promiscuity, which requires further investigation.

The wealth quintile, knowledge on HIV test kits, ARV drugs, and HIV knowledge score were strong predictors. Our findings that the women in the richer quintile had lower odds of having HIV stigma are consistent with the findings from the 64-country study.^[28]^ In addition, the findings from the current study indicated higher odds of HIV stigma among rural residents, aligning with previous research: 72% of rural women in Ethiopia had HIV stigma.^[29]^ Our findings also align with those of Sallam *et al*. (2022). In their study, there was a significant relationship between insufficient HIV knowledge and negative attitudes toward PLWHA among the general population.^[30]^ Perhaps, limited knowledge or cultural perspectives (i.e., linking HIV with promiscuousness) on HIV transmission may create misconceptions and HIV stigma. Similarly, the present study findings revealed that those who were employed had lower odds of having HIV stigma. It is possible that the women in employment have exposure to the HIV information or awareness campaign, leading to reduced HIV stigma. This requires further investigation.

The positive relationship between HIV stigma and knowledge of HIV test kits and ARV drugs represents novel findings. Respondents who heard about HIV test kits, despite never getting tested, were aware of ARV drugs, and those who knew about drugs that prevent MTCT had lower odds of having HIV stigma. Further research on these areas may help the researchers and decision makers to better understand the relationship between HIV stigma and knowledge of HIV testing/ARV drugs, as these are new findings.

Our findings indicate that women aged 15–24 years who lack awareness of HIV risk reduction and hold misconceptions about HIV transmission have higher odds of having HIV stigma toward PLHIV compared to their counterparts. Specifically, our results indicate that those with an HIV knowledge score of one or higher suggest that awareness campaigns about HIV transmission and risk reduction should be consistently conducted to reach this younger population. Furthermore, our findings indicate that rural, young married women aged 15–24 years had higher levels of HIV stigma toward PLHIV. Maybe these rural residents had limited access to HIV information. In addition, it is possible that traditional taboos in rural societies discourage young married women from discussing HIV, further hindering their understanding of stigma’s impact. Overall, the findings from this study highlight an urgent need for HIV stigma reduction at all levels within the general population, especially targeting rural young married residents. Addressing the issue of HIV stigma will contribute to achieving global goals: Sustainable Development Goal (SDG) 3 (good health and well-being) and subsequently help to reach SDG 6 (promoting gender equality) and SDG 10 (mitigating inequalities).

### Strengths and Limitations of the study

The main strength of this study is the use of the most recent nationally representative data, reflecting the current situation. However, this study has some limitations. First, this study used cross-sectional data, making it impossible to establish causal relationships. Second, self-reported data might pose a recall or social desirability bias. Third, we acknowledge that the present study’s use of only two variables may not fully and comprehensively capture the multi-dimensional nature of stigma. However, these two variables were aligned with the UNAID’s GAM indicator 6.1 on discriminatory attitudes.^[11]^ Fourth, for HIV knowledge on transmission and risk reduction, data are only available for young women aged 15–24, highlighting a need to include all age groups in future research. Finally, although we adjusted potential confounders using multivariable logistic regression, there could be other confounders that we did not include in our analysis. This requires further exploration. Despite all these limitations, our study’s findings make a significant contribution to the decision makers and future researchers.

## CONCLUSION AND GLOBAL HEALTH IMPLICATIONS

This study highlighted a higher percentage of HIV stigma in the study population. As stated elsewhere, stigma could pose a lot of burden and stress for individuals. Such stress can lead to engaging in HIV risks and causing them to delay HIV testing, treatment, and care. To prevent such delays, current stigma reduction strategies should be reviewed and revised. The present study findings can be used to advocate for decision-makers to put extra effort into reducing stigma. Future public health research should explore the underlying mechanisms of the stigmatizing attitudes and discriminatory behaviors among the general population.

### Key Messages

(1) What is already known. There is a relationship between HIV knowledge, especially transmission and prevention, and stigma. The limited knowledge on transmission and prevention among young women suggests that continued and consistent HIV awareness campaigns should be done.

(2) What is new: The present study’s finding of a positive relationship between the stigma and knowledge related to HIV test kits and ARV drugs is new. Individuals with such knowledge exhibited lower odds of HIV stigma toward PLHIV and suggested that there should be increased educational events to spread this knowledge among the general population. (3) These findings highlighted an urgent need to address societal stigma among Filipino women so that they can get an HIV test early (if needed) and link to care and treatment, which are critical for HIV epidemic control.

## Acknowledgments

None.

## COMPLIANCE WITH ETHICAL STANDARDS

### Conflicts of Interest

The author declares no competing interests.

### Financial Disclosure

Nothing to declare.

### Ethics Approval

Not applicable. We used publicly available de-identified data.

### Declaration of Patient Consent

Patient’s consent is not required as there are no patients in this study.

### Use of Artificial Intelligence (AI)-Assisted Technology for Manuscript Preparation

The authors confirm that there was no use of artificial intelligence (AI)-assisted technology for assisting in the writing or editing of the manuscript and no images were manipulated using AI.

### Disclaimer

None.
